# Numerical Simulation of Thermal Cycling and Vibration Effects on Solder Layer Reliability in High-Power Diode Lasers for Space Applications

**DOI:** 10.3390/mi16070746

**Published:** 2025-06-25

**Authors:** Lei Cheng, Huaqing Sun, Xuanjun Dai, Bingxing Wei

**Affiliations:** 1 College of Mechanical and Control Engineering, Guilin University of Technology, Guilin 541000, China; sunhuaqing@jcgjd.org.cn (H.S.); weibingxing2023@163.com (B.W.); 2 Jincheng Research Institute of Opto-Mechatronics Industry, Jincheng 048000, China; 3 Shanxi Key Laboratory of Advanced Semiconductor Optoelectronic Devices and Integrated Systems, Jincheng 048000, China

**Keywords:** space environment, high-power laser diode, solder layer, thermal cycling, random vibration, life prediction

## Abstract

High-power laser diodes (HPLDs) are increasingly used in space applications, yet solder layer (SL) reliability critically limits their performance and lifespan. This study employs finite element analysis to evaluate SL failure mechanisms in microchannel-cooled HPLDs with two packaging configurations under thermal cycling and vibration. Based on the Anand constitutive model, contour plot analysis revealed that the critical stress–strain regions in both SLs were located at their edges. The stress–strain values along the X-axis of the SLs exceeded those in other axial directions, and SL failure would preferentially initiate from the edges along the cavity length direction. During random vibration analysis with excitation applied along the Z-axis, the equivalent stresses in both SLs exceeded X-/Y-axis levels. However, these values remained far below their yield strengths, indicating that only elastic strain and high-cycle fatigue occurred in the SLs. The calculated thermal fatigue lives of the two SLs were 2851 cycles and 5730 cycles, respectively. Their random vibration fatigue lives were determined as 5.75 × 10^7^ h and 8.31 × 10^7^ h. Using damage superposition under combined thermal-vibration loading, the total fatigue lives were predicted as 14,821 h and 29,786 h, respectively, with thermal cycling-induced damage dominating the failure mechanism.

## 1. Introduction

Laser diode (LD) has displayed rapid development since 1962 when the world’s first LD came into being, with increasing types and expanding application scope [1,2,3,4,5]. As a class of laser-generation devices with semiconductor materials as the operating substance, LD has gradually become one of the indispensable photoelectric devices in modern science and technology following decades of development. HPLDs, due to their small volume and mass, high electro-optical conversion efficiency, long service life, and wide wavelength covering range, have been extensively applied in such fields as industry, military, communication, optical storage, and laser medicine in recent years [6,7]. With the development of semiconductor technology, LDs have become the research object of many scholars, and a lot of progress has been made in the basic theory and physical mechanism research, material research, manufacturing process research, etc., which has prompted the development of the HPLDs towards high power, high efficiency, and miniaturization.

The SLs are an essential part of the packaging structure of the HPLDs. The SL serves as the medium that connects the LD chip to the heat sink (HS), N-foil, insulating layer, and other layer structures. The SL plays several key roles in the operation of an HPLD. The SL assumes the task of electrical connection so that the HPLD can transmit electrical signals; it also assumes the task of mechanical connection and can play the role of a stress buffer, the LD chip fixed in the HS, so that it cannot fall off; it also provides a space to make the package chip to form a heat dissipation path, reducing the impact of the heat generated on the chip. Once the SL has a problem, it will inevitably lead to the HPLD as a whole not working properly. In the field of electronic packaging reliability, interface (this paper for the SL) reliability engineering has always occupied the core research position. Therefore, how to improve the environmental adaptability of the SL of the HPLD has become the focus of improving the reliability of the HPLD in harsh environments.

**Figure 1:**
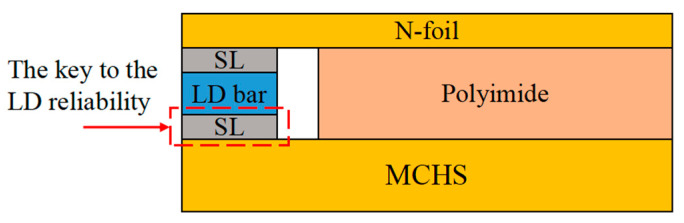
The critical SL affecting laser reliability.

In laser packaging, the SLs are used for connection in multiple places. According to the research results of Ephraim Suhir [8], Zhangpu [9], and others [10,11,12], it is determined that the SL connecting the chip and the HS play a key role in the reliability of the HPLD, that is, the marked place in Figure 1. Ephraim Suhir et al. [8] used the finite element method to systematically simulate the SL of the HPLD. They simulated the steady-state and transient thermal behaviors of conduction-cooled HPLDs in continuous wave (CW) mode and explained the important influence of SL reliability on the overall performance and service life of HPLDs from the perspective of thermal stress: On the one hand, the SL has low yield strength and few fatigue cycles, making it the “weak link” connecting the HS and the chip. Under cyclic thermal stress, the SL usually starts to age and fail before other structures; on the other hand, the SL provides a “buffer zone” between the chip and the HS. By matching the coefficient of thermal expansion (CTE), it offsets part of the interaction between the chip and the HS under thermal stress, reduces the thermal deformation of the chip, and prolongs the service life of the device. In addition, the model designed in this paper can calculate the transient peeling stress and shear stress of the SL in CW mode. Therefore, in order to reduce the calculation amount and simplify the name, unless otherwise specified, the term “SL” in the following text refers to the SL connecting the bar chip and the microchannel heat sink (MCHS).

Applied to the space environment LD in the process of processing, loading and adjusting, transportation, launching and orbiting, etc., the LD will be subjected to a variety of complex, harsh environmental effects, such as high temperatures, low temperatures, high- and low-temperature cycling, acceleration overload, random vibration, and so on. Therefore, the environmental adaptability of the LD has put forward higher requirements. Failure mechanism analysis shows that temperature loading (55%) and mechanical vibration (20%) together constitute the dominant factor in the dual-stress-coupled failure mechanism, while the remaining 25% of the failures are attributed to other environmental stress compounding effects (humidity, barometric pressure perturbation, etc.). This failure distribution characteristic confirms [13,14,15,16,17], at a statistical level, the engineering necessity of establishing a reliability assessment system based on the accelerated thermal–vibration biaxial degradation effect. The environmental adaptability requirements [18] cover 13 types of environmental stress conditions (thermal cycling, random vibration, etc.), which would result in a waste of resources if full-scale tests of the same intensity were used. According to the statistical analysis of failure inducers of electronic devices [19], thermal cycling and random vibration are located in the top two. Therefore, these two main triggers are selected as the loading conditions for environmental tests to be studied in depth in this paper. W. Wright et al. [20] conducted reliability testing of commercial high-power fiber-coupled LDs for space applications, performing mechanical, vibration, and thermal cycling, radiation testing, and destructive part analysis and verifying that they met the 5000 h service life requirement through a 500 h accelerated life test. The test device was stable under a 20 krad radiation dose, −40 to 60 °C thermal cycling, and 20 g vibration but failed due to a package defect during constant acceleration testing. Pol Ribes-Pleguezuelo et al. [21] focused on the small diode-pumped laser of the Raman Laser Spectrometer of the Exomars mission to evaluate soldering and bonding through thermal cycling, random vibration, sine vibration, and shock testing. Vibration and shock tests evaluated the performance stability of the laser under solder and adhesive assembly processes. The adhesive-assembled laser showed no significant damage after the vibration and shock tests, but the output power continued to drop during thermal cycling, the optical performance deteriorated, and the laser resonance cavity showed gradual misalignment. The low-stress weld-assembled lasers showed good stability of the main optical parameters after functional thermal cycling and mechanical and ion radiation tests, with no obvious output power degradation or damage.

In the field of military electronic equipment reliability verification, MIL-STD-810F pointed out that [18] the use of simulation environment test methods in the product development period not only can significantly shorten the test cycle and reduce costs but also ensure that the product meets the adaptability of the actual service environment requirements. Most of the existing electronic packaging reliability research focuses on the traditional microelectronics packaging field of various types of solder joint failure behavior and for optoelectronic integrated devices—the HPLD chip SL, especially, in the multi-field coupling effect of the interface reliability of the systematic research is relatively scarce.

In this paper, the three-dimensional physical model of two kinds of microchannel packaged HPLDs is established, and the reliability of the SL of the two kinds of packaged HPLDs is compared and studied under the effects of thermal cycling, random vibration, and thermal–vibration coupling. Under the loading conditions of thermal cycling and random vibration, the fatigue failure position of the SL is studied, the maximum equivalent stress and strain distribution at this position are analyzed, and the fatigue life of the SL is predicted under the combined action of thermal vibration and vibration coupling loading.

## 2. Numerical Model and Theoretical Analysis

### 2.1. Physical Geometric Model

**Figure 2:**
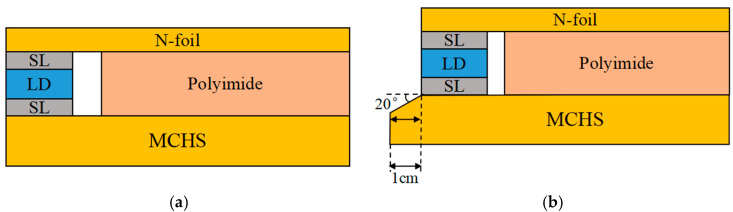
Schematic diagram of the LD packaging. (**a**) Before optimization and (**b**) after optimization.

**Figure 3:**
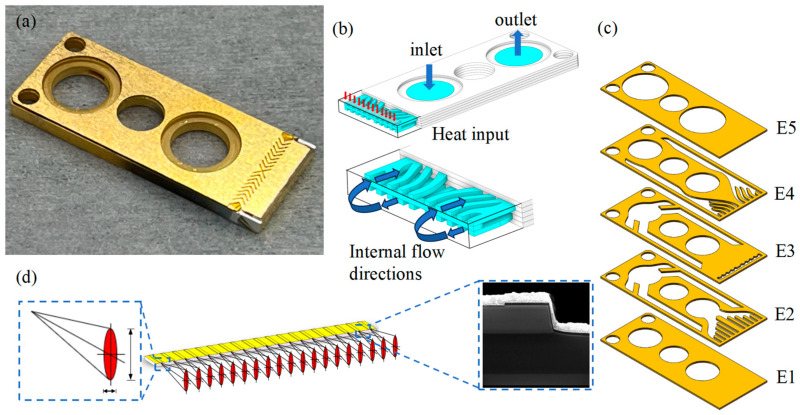
Schematic diagram of the LD. (**a**) Physical diagram of the LD in the MCHS package; (**b**) schematic diagram of the MCHS heat dissipation; (**c**) internal structure of the MCHS, E1, E2, E3, E4, and E5 are the Bottom Layer, Inlet Layer, Separating Layer, Outlet Layer, and Top Layer, respectively; and (**d**) the LD bar and its local magnification.

The author’s research team [22] proposed an optimized MCHS packaging form for space environment in a previous study. The optimized package form improves the heat transfer efficiency and heat dissipation performance of the HPLD, and the maximum temperature of the HPLD was reduced from 52.44 °C to 41.23 °C. The optimized package form has good overall performance. This paper presents a comparative study of the HPLDs using pre-optimization and post-optimization package forms. The cross-sectional diagram of the HPLD package structure before optimization is shown in Figure 2a. Schematic cross-section of the HPLD package structure after optimization is shown in Figure 2b. As shown in Figure 3a, lasers were equipped with a microchannel water-cooled packaging structure, which consisted of an MCHS, SLs, an LD bar, an insulating film, and a copper sheet. The bar was packaged with P surface adown and welded at the front end of the microchannel water-cooled HS with SL. Distilled water flowed into the internal MCHS from the water inlet, fully exchanged heat with the copper wall, took away the heat generated by the bar during operation, and finally flowed out from the water outlet, as shown in Figure 3b. The parameter of the MCHS was 27 mm × 10.8 mm × 1.5 mm, and the MCHS was composed of five layers of stitch-welded oxygen-free high-conductivity copper, as shown in Figure 3c. The LD bar studied in this paper had a bar wavelength of 808 nm, a ridge width of 150 μm, a filling factor of 30%, a cavity length of 1000 μm, and 19 light-emitting points. At 25 °C, the injection current was 50 A, the maximum output power was 55.31 W, the electro-optical conversion efficiency was 58.74%, the slope efficiency was 1.21 W/A, and the divergence angles of the slow and fast axes of the laser die were less than 10° and less than 39°, as shown in Figure 3d. The properties of each material are shown in Table 1.

### 2.2. Anand Viscoplastic Principal Model

In this study, Anand’s intrinsic model with multi-mechanism coupling properties was selected for mechanical characterization of indium solder under thermal loading for its viscoplastic behavior characteristics [23].

The core equations of the Anand model consist of the flow equation and the evolution equation:

The flow equation describes the relationship between strain rate and stress:

(1) $$\varepsilon_{p}=Asinh{\left(\frac{\xi \sigma }{s}\right)}^{1/m}exp\left(-\frac{Q}{RT}\right)$$

where $\varepsilon_{p}$ is the inelastic strain rate, A is a constant, Q is the activation energy, R is the gas constant, T is the absolute temperature, ξ is the stress factor, and m is the strain rate sensitivity factor. All temperature parameters used in subsequent calculations based on this formula have been uniformly converted to the SI unit Kelvin (K) to ensure its correct application.

The evolution equation describes the evolution of the deformation impedance S:

(2) $$S=\left[{\left|1-\frac{S}{S^{*}}\right|}^{a}sign\left(1-\frac{S}{S^{*}}\right)h_{0}\right]\varepsilon_{p}$$

(3) $$S^{*}=\hat{S}{\left[\frac{\varepsilon_{p}}{A}exp\left(\frac{Q}{RT}\right)\right]}^{n}$$

where a and $h_{0}$ are the material strain hardening parameters, $S^{*}$ is S the saturation value, and $\hat{S}$ is the coefficient of deformation resistance saturation value.

The nine parameters in the Anand model: A, Q/R, ξ, $S^{\prime }$, n, m, a, $h_{0}$ and initial deformation resistance $S_{0}$ can be obtained from uniaxial tensile experiments at several sets of different strain rates and temperatures. The Anand parameters for indium used in this paper are shown in Table 2.

### 2.3. Fatigue Life Prediction Model

#### 2.3.1. Fatigue Life Prediction Model Under Thermal Loading

For the fatigue life prediction of failure-prone structures such as solder balls, solder joints, and SLs in chip packaging, experts and scholars have proposed a variety of models and methods. Among them, the modified Coffin–Manson model proposed by Engelmaier is one of the classic theories, which is widely used to predict the life of the SL under fatigue load based on the level of plastic deformation and by studying the stress level of the material, strain amplitude, and other factors.

The Coffin–Manson fatigue life prediction model was improved by Engelmaier et al. to be mathematically expressed as [24]

(4) $$N_{f}=\frac{1}{2}{\left(\frac{\Delta \gamma_{p}}{2\varepsilon_{f}}\right)}^{\frac{1}{c}}$$

(5) $$c=-0.442-6\times 10^{-4}T+1.74\times 10^{-2}\times \ln\left(1+f\right)$$

(6) $$\Delta \gamma_{p}=\sqrt{3}\Delta \varepsilon$$

where $\Delta \gamma_{p}\;$ is the equivalent shear strain amplitude, Δε is the equivalent plastic strain amplitude, c is the modified fatigue toughness index, f is the frequency of cycling, T is the temperature average, $\varepsilon_{f}$ is the fatigue toughness coefficient, and its empirical value = 0.325.

#### 2.3.2. Fatigue Life Prediction Model Under Random Vibration Loading

For the fatigue failure of welded layers induced by random vibration loads, the current life prediction methods mainly form two types of analytical frameworks in the frequency domain and time domain. In this study, an evaluation system based on the energy analysis method in the frequency domain is constructed, and the prediction system is constructed by integrating Manson’s high perimeter fatigue theory, the three-band technique, and the linear fatigue damage accumulation criterion.

(1) Manson’s high-frequency fatigue equation

Its core Manson–Coffin relational equation originated from a large number of fatigue test studies, and the mathematical characterization of the total fatigue life was finally derived by superimposing the plastic strain life curve with the elastic strain life curve [25]:

(7) $$\frac{∆\varepsilon_{t}}{2}=\frac{∆\varepsilon_{el}}{2}+\frac{∆\varepsilon_{pl}}{2}=\frac{\sigma_{f}^{\prime }}{E}{\left(2N_{f}\right)}^{b}+\varepsilon_{f}^{\prime }{\left(2N_{f}\right)}^{c}$$

where $∆\varepsilon_{t}$ is for the total strain amplitude; $\;∆\varepsilon_{el}$ is for the elastic strain amplitude; $∆\varepsilon_{pl}$ is for the plastic strain amplitude; $\sigma_{f}^{\prime }$ is for the fatigue strength coefficient; E is for the material elastic modulus; b is for the fatigue strength index; $\varepsilon_{f}^{\prime }$ is for the fatigue plasticity coefficient; and c is for the fatigue plasticity index.

The generalized slopes of the elastic and plastic terms are −0.12 and −0.6, respectively, which can be obtained by substituting the two terms into the following equation:

(8) $$∆\varepsilon_{t}=3.5\frac{\sigma_{u}}{E}N_{f}^{-0.12}+\varepsilon_{f}^{0.6}N_{f}^{-0.6}$$

where $\sigma_{u}$ is the tensile ultimate strength of the material.

When elastic strain occurs, Manson’s empirical equation for high week fatigue is

(9) $$∆\varepsilon_{t}=∆\varepsilon_{el}=3.5\frac{\sigma_{u}}{E}N_{f}^{-0.12}$$

(10) $$\varepsilon =\frac{∆\varepsilon_{t}}{2}=3.5\frac{\sigma_{u}}{2E}N_{f}^{-0.12}$$

The SL material is indium, taking its modulus of elasticity at room temperature as 13 GP and its tensile ultimate strength as 4.5 MPa, and the empirical formula for Manson’s high week fatigue is

(11) $$\varepsilon =0.00061N_{f}^{-0.12}$$

(2) Three-band technology

The reliability of the three-band technique as an effective method for stochastic vibration fatigue analysis has been verified by a large amount of experimental data. The technique is based on the assumption that the vibration acceleration obeys a Gaussian distribution, where the vertical axis represents the probability density and the horizontal axis characterizes the ratio of the instantaneous acceleration to the Root Mean Square (RMS) acceleration response value. The probability distribution of the vibration response is characterized by the typical three zones: The ±1σ interval covers 68.27% probability, the ±2σ interval accumulates to 95% probability, and the ±3σ interval reaches the highest probability share of 99%.

The fatigue damage index accumulated by the object in random vibration conditions during the next hour can be calculated from Equations (12)–(14).

(12) $$n_{1}=N_{0}^{+}T_{v}\left(3600s/h\right)\left(0.6831\right)$$

(13) $$n_{2}=N_{0}^{+}T_{v}\left(3600s/h\right)\left(0.2710\right)$$

(14) $$n_{3}=N_{0}^{+}T_{v}\left(3600s/h\right)\left(0.0433\right)$$

The probability of an instantaneous acceleration occurring is 99.7%, which tends to be close to 1. Therefore, the maximum acceleration level is usually considered to be horizontal in engineering practice.

Expression (15) is for the number of positive zero passes ($N_{0}^{+}$) of the displacement [26]:

(15) $$N_{0}^{+}=\frac{1}{2\pi }{\left[\sum \frac{P_{i}f_{i}Q_{i}^{2}}{\Omega_{i}^{2}}/\sum \frac{P_{i}f_{i}Q_{i}^{2}}{\Omega_{i}^{4}}\right]}^{\frac{1}{2}}$$

(3) Fatigue damage accumulation theory

When the material is subjected to alternating stresses exceeding its fatigue threshold, each load cycle triggers a progressive accumulation of microstructural damage. This damage mechanism is irreversible, and its cumulative effect will lead to material failure when it reaches the critical threshold, which constitutes the core mechanism of fatigue damage evolution theory. At present, Miner’s linear cumulative damage criterion is commonly used as the classical model in engineering practice, which realizes the life prediction through the linear superposition of damage degrees.

**Figure 4:**
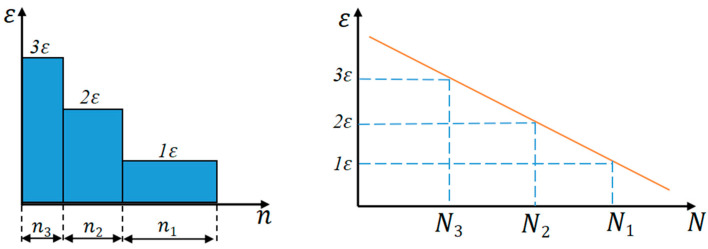
Random loads of different magnitudes.

For random vibration, the time-varying amplitude loads on the structure need to be handled by the load spectrum discretization method. The three-band hierarchical technique shown in Figure 4 discretizes the continuous random vibration signal into three characteristic load intervals based on the principle of stress amplitude hierarchy, and this quantitative method provides an effective engineering solution path for fatigue assessment of variable amplitude loads.

(16) $$D_{v}=\sum \left(n_{i}/N_{i}\right)\left(i=1,2,3\right)$$

(17) $$D_{v}=\frac{n_{1}}{N_{1}}+\frac{n_{2}}{N_{2}}+\frac{n_{3}}{N_{3}}$$

#### 2.3.3. Fatigue Life Prediction Model Under Thermal–Vibration Coupled Loading

**Figure 5:**
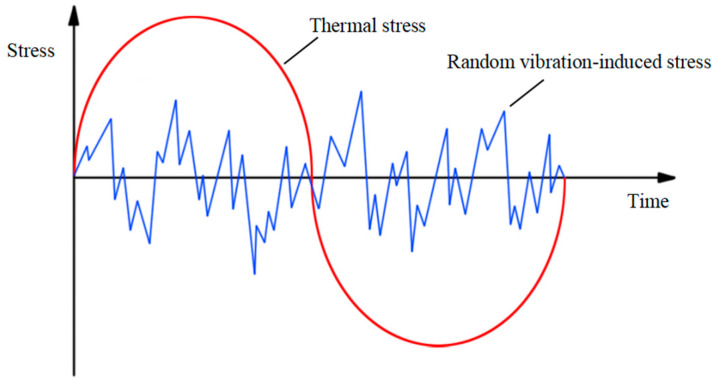
Stress diagram caused by the combination of thermal cycling and random vibration.

During the entire life cycle of an HPLD, the compound environmental stresses during transportation and use can cause critical interface failures. Especially in aerospace and military high-reliability application scenarios, multi-physical field coupling effects (mechanical vibration and thermal cycle synergy) in the SL region will induce a synergistic crack expansion effect. The composite strain energy generated by such multi-axial stresses significantly shortens the fatigue life cycle of the SL through the damage accumulation mechanism, which ultimately leads to the loss of structural integrity of the SL. Figure 5 shows a schematic diagram of the stresses and strains generated by the combined action of random vibration and thermal cycling on the SL.

In this multi-field coupling environment, the composite load effect can be quantitatively modeled based on the principle of linear damage superposition. The damage coupling equation established using the Miner criterion can transform the nonlinear damage process triggered by mechanical vibration and thermal cyclic loading into linearly superimposable damage coefficients so as to construct a life prediction model of the SL under the action of multiple physical fields.

(18) $$D_{total}=D_{th}+D_{v}=\frac{n_{th}}{N_{th}}+\frac{n_{v}}{N_{v}}=n_{th}\left(\frac{f_{v}/f_{th}}{N_{v}}+\frac{1}{N_{th}}\right)$$

where $D_{v}$ is the fatigue damage factor caused by vibration fatigue; $D_{th}$ is the fatigue damage factor caused by thermal cycling fatigue; $n_{th}$ is the actual thermal cycling cycles; $f_{v}$ is the structural vibration frequency; and $f_{th}$ is the thermal cycling frequency.

Under the composite loading environment, the random vibration will always have the process of converting from the frequency domain to the time domain and then coupling with the thermal cycle. Therefore, when the thermal vibration coupling is considered comprehensively, the energy dissipation path of the SL under the action of nonlinear damage mechanism can be obtained, and its theoretical solution is shown in the engineered life assessment model shown in Equation (19).

(19) $$N_{f}=\frac{D}{\left(\frac{f_{v}/f_{th}}{N_{v}}+\frac{1}{N_{th}}\right)}$$

where $N_{f}$ is equivalent to the number of thermal fatigue cycles.

## 3. Simulation Evaluation of the SL Reliability Under Thermal Cyclic Loading

### 3.1. Analyzing Processes

Finite element software (ANSYS2023R1) was applied to establish the three-dimensional model of the HPLD. In order to reduce the amount of operation and improve the speed of operation, without affecting the simulation results, the HPLD was reasonably simplified, only retaining the structures such as the bar, the SL, the MCHS, etc. and ignoring the other structures that have a small impact on heat dissipation. The mapping method was used to divide the mesh, and the mesh encryption was carried out for the key areas of concern such as the bar and the interconnection interface below, with a mesh size of 1 × 10^−4^ m, and the mesh size of the MCHS part of the mesh is 5 × 10^−4^ m. Combined with the space environment, the conditions were set up, and the microchannel water-cooled encapsulation of the MCHS was set to be fixed at the bolt holes on the microchannel, and the temperature was cyclically loaded to the whole model.

The grid sensitivity was verified. When the number of grids was 17,785, the error between the result and the number of grids was less than 1%. It is proven that the selected grid size has good accuracy and a fast running speed, as shown in Table 3.

**Figure 6:**
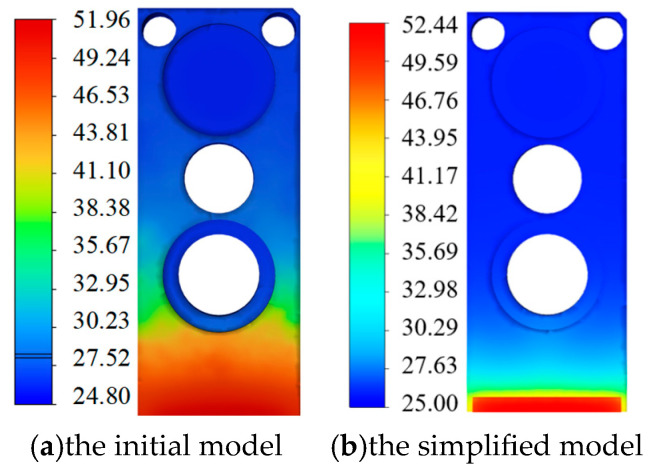
Temperature distribution cloud diagrams before and after model simplification.

The thermal simulation results before and after the model simplification are shown in Figure 6a and Figure 6b, respectively. It can be seen that the difference in the maximum temperature between the two is 0.48 °C, which has a relatively small influence on the results.

**Figure 7:**
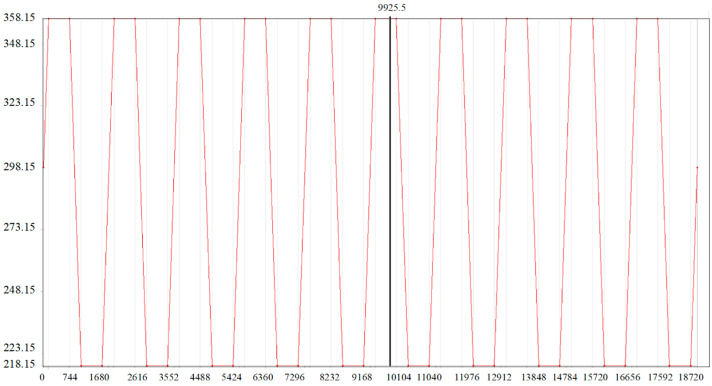
Temperature cycling load.

Based on the NASA Goddard Space Flight Center issued by the space flight environment with the HPLD array qualification guide [26] and MIL-STD-883 standard [27] to develop the thermal cycle load, the temperature range is 218.15 K to 358.15 K. The initial temperature was set to 298.15 K, the rate of change in the temperature was 25 K/min, the lowest temperature and the highest temperature were maintained for 10 min, the cycle was 31.2 min, the model 10 cycles were carried out, and the temperature loading was carried out in accordance with Figure 7.

### 3.2. Results and Analysis

**Figure 8:**
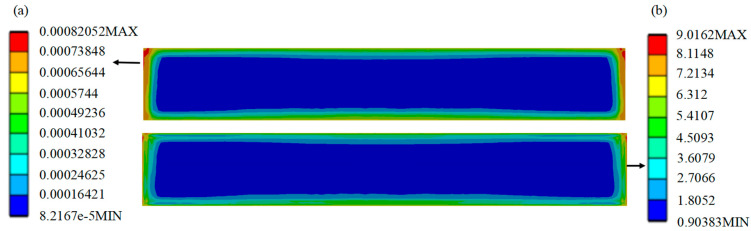
Equivalent stress and strain distribution cloud diagram of the SL before optimization.

**Figure 9:**
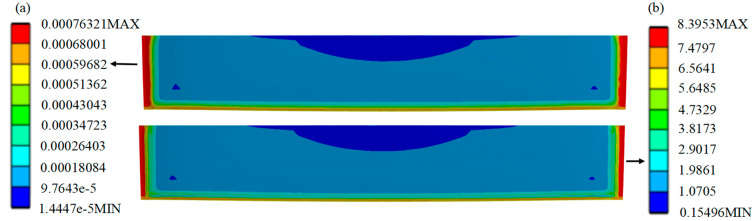
Equivalent stress and strain distribution cloud diagram of the SL after optimization.

Comparison of the performance of the SL of the HPLD using the MCHS package before and after optimization is as follows: The strain distribution cloud (Figure 8a and Figure 9a) is shown, and the stress distribution cloud (Figure 8b and Figure 9b) is shown. Before optimization, the maximum plastic strain is concentrated in the four corners of the SL, in which node 4505 is confirmed as the failure hazard point by thermal cycle analysis, and the corresponding maximum stress value reaches 9.016 MPa; after optimization, the hazardous area is shifted to the edge of the SL, and node 4518 becomes the new strain concentration point, and the maximum stress value is reduced to 8.40 MPa. The data show that the maximum stress in the SL is reduced to 0.621 MPa after optimization of the structure. The data shows that the maximum stress in the SL is reduced by 0.621 MPa after structural optimization, which effectively improves the reliability of the package.

During the thermal cyclic loading process, the multi-axial stress coupling effect of the SL is triggered by the gradient distribution of the thermal expansion coefficients of the SL, the bar, the MCHS, the insulating film, and the copper foil. For the numerical analysis of this complex stress field, the Von Mises yield criterion becomes the preferred solution.

In the Von Mises yield criterion [28], the equivalent stress is described in terms of components of the stress tensor as

(20) $$\sigma_{s}=\sqrt{\frac{1}{2}\left[{\left(\sigma_{1}-\sigma_{2}\right)}^{2}+{\left(\sigma_{2}-\sigma_{3}\right)}^{2}+{\left(\sigma_{3}-\sigma_{1}\right)}^{2}\right]}$$

According to the Von Mises criterion, the specific energy of shape change, when flow occurs under unidirectional stretching, is calculated by making $\sigma_{1}=\sigma_{s}$, $\sigma_{2}=\sigma_{3}=0$, and the shear yield strength of indium K = 10 MPa:

$$\sigma_{s}=\sqrt{\frac{1}{2}\left[{\left(\sigma_{1}-\sigma_{2}\right)}^{2}+{\left(\sigma_{2}-\sigma_{3}\right)}^{2}+{\left(\sigma_{3}-\sigma_{1}\right)}^{2}\right]}=\sqrt{3}K=1.732\times 10=17.32\;MPa$$

The Von Mises stress at the point of maximum stress is much smaller than the equivalent stress so that thermal cyclic loading does not have a destructive effect on the SL.

For the HPLD using optimized pre- and post-MCHS packages, the hazardous areas of stress and strain on the SL are located at the edges of the SL, showing a diffusion from the middle to both sides. The SL is characterized by a significant anisotropic stress distribution: The axial stress (Z-direction) shows a quasi-uniform distribution, while a gradient stress field is formed in the transverse direction (X-direction). Based on the coupling of the stress field characteristics in the symmetry axis region and the thermal deformation mechanism, a significant stress gradient distribution is observed inside the SL: The equivalent stresses in opposite directions in the neighboring symmetry axis cancel each other out to form a low-stress region; with the increase in the distance from the axis, an isotropic stress superposition phenomenon is gradually observed at the edge of the SL, with an exponential decay in the canceling efficiency. Under thermal cyclic loading, the in-plane shear deformation caused by the difference in thermal expansion coefficients between the bar and the SL material shows a non-uniform distribution—the edge region produces significant warpage strain concentration due to the weak geometric constraints, while the center region is subjected to bi-directional constraints for relatively coordinated deformation. These reasons lead to an increasing trend of stress–strain in the SL along the X-axis from the center to both sides. Based on this stress field evolution law, cracks preferentially sprout in the high-stress singularity region and then expand along the direction of the maximum principal stress gradient (in the cavity length direction), eventually forming a penetrating failure path.

**Figure 10:**
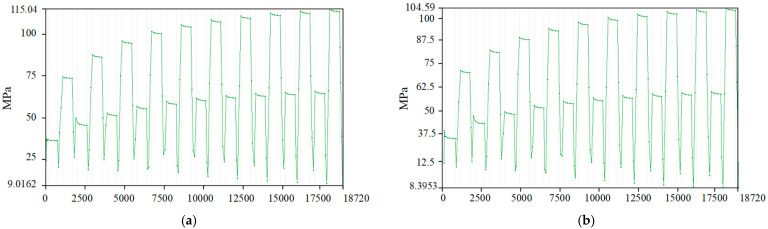
Equivalent stress curve diagram of the SL. (**a**) Packaging before optimization and (**b**) packaging after optimization.

Figure 10 shows the equivalent stress plots of the SL of the HPLD with the MCHS package before and after optimization, respectively. As seen from the data curves, the stress change of the SL shows a synchronous fluctuation law with the temperature cycle. When the temperature remains stable, a small release of internal material stress occurs; while in the temperature rise and fall stage, the stress level all climbs up, and the stress increase generated by the cooling process is more significant than that of heating. This temperature–stress linkage reveals the sensitive response of the material to changes in the thermal environment.

### 3.3. Fatigue Life Prediction Under Thermal Loading of the SL

For the entire HPLD’s SL, the edge region of the SL is the most likely to fail; through the above analysis, it can be seen that in the case of thermal cyclic loading, the deformation of the SL is mainly due to the mismatch of the coefficients of thermal expansion between the materials caused by plastic deformation. Therefore, the life prediction model in this paper is based on plastic deformation to calculate the fatigue life of the SL. After the simulation solution is stabilized, the value of the equivalent plastic strain range in the hazardous region of the SL is the difference in the maximum value of the equivalent plastic strain.

The equivalent plastic shear strain range of the HPLD’s SL encapsulated before optimization is

$${∆\gamma }_{p}=\sqrt{3}∆\varepsilon =0.009927$$

Substituting the data into the formula gives the following:

$$T=\frac{-55+85}{2}=15$$

f=24×60×60÷18720≈4.6

$$c=-0.442-6\times 10^{-4}\times 15+1.74\times 10^{-2}\times \ln\left(1+4.6\right)=-0.42$$

$$N_{f}=2851$$

Similarly, the life of the optimized packaged HPLD’s SL under thermal cyclic loading conditions is 5730 cycles.

The thermal fatigue life of the SL before and after optimization is 2851 cycles and 5730 cycles, respectively. The thermal fatigue life prediction shows that the life of the optimized packaged HPLD’s SL is twice as long as the life of the original packaged HPLD’s SL. The optimized and improved packaged HPLD’s SL life has been significantly improved, which can better cope with the harsh service environment and ensure the thermal reliability of HPLS in the space environment.

## 4. Simulation Evaluation of the SL Reliability Under Vibration Loading

### 4.1. Analyzing Processes

**Figure 11:**
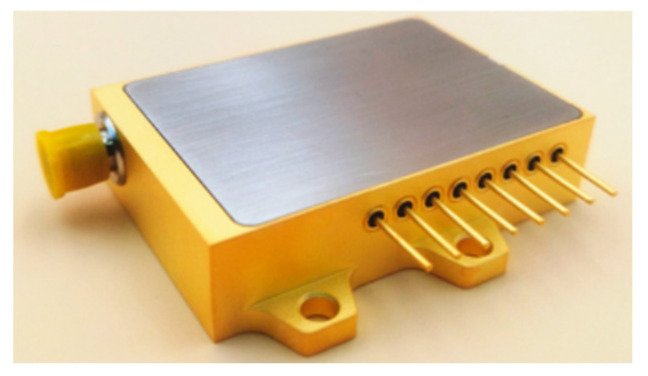
LD module.

When performing modal analysis, continuing to use only the HPLD for vibration analysis will result in the loss of modes and a large error in the subsequent calculations, so this subsection uses the LD module for the simulation and analysis; the model is shown in Figure 11. The HPLD is located inside the module, and the laser is consistent with the above. The LD module shell material is 6061-T6 aluminum alloy, with dimensions of 80 mm × 40.5 mm × 13 mm, and the physical parameters are shown in Table 4.

### 4.2. Results and Analysis

(1) Modal analysis

**Figure 12:**
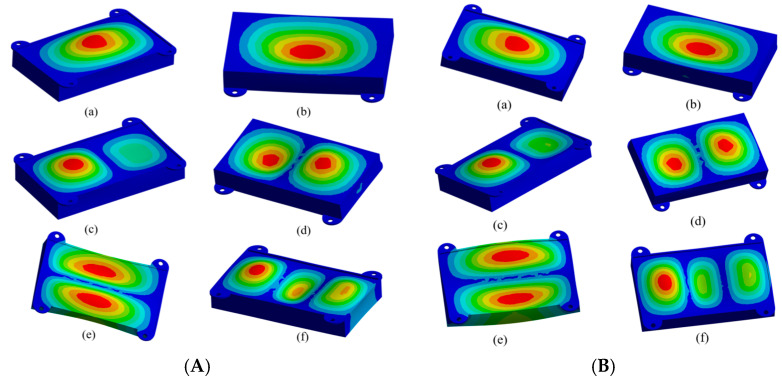
Mode shapes of the first six vibration modes of the LD module. (**A**) Before optimization and (**B**) after optimization.

According to the vibration theory, the modal study of the LD module is carried out by using Subspace for modal analysis. Constraining the degrees of freedom of the nodes attached to the bolt holes, the first six orders of modal frequencies are shown in Table 5, and the modal analysis of the LD module is carried out to obtain the first six orders of vibration patterns of the LD module before and after the optimization, as shown in Figure 12A,B, and the 1st–6th orders of modal patterns are shown in Figure 12a–f, respectively.

The intrinsic frequency is an inherent property of the structure itself, independent of external excitation. The intrinsic frequency of the structure obtained from the modal analysis can avoid the resonance of the structure during the design process. Comparison of Figure 12A,B shows that the vibration pattern before and after optimization is almost the same, which indicates that the optimization improvement will not adversely affect the intrinsic frequency of the LD.

(2) Random vibration analysis

In the study of structural dynamics, the spectral analysis method can effectively solve the dynamic response problem under broadband excitation by combining the structural modal characteristics with the excitation spectral features. The random vibration excitation to which the LD module is subjected is an acceleration excitation, which is loaded through the bolted joints in the finite element model. According to the test guidelines published by the Goddard Space Flight Center, the power spectral density analysis function is used to input the acceleration power spectral density (PSD) as shown in Table 6, and each of the three directions of X, Y, and Z axes need to be tested.

**Figure 13:**
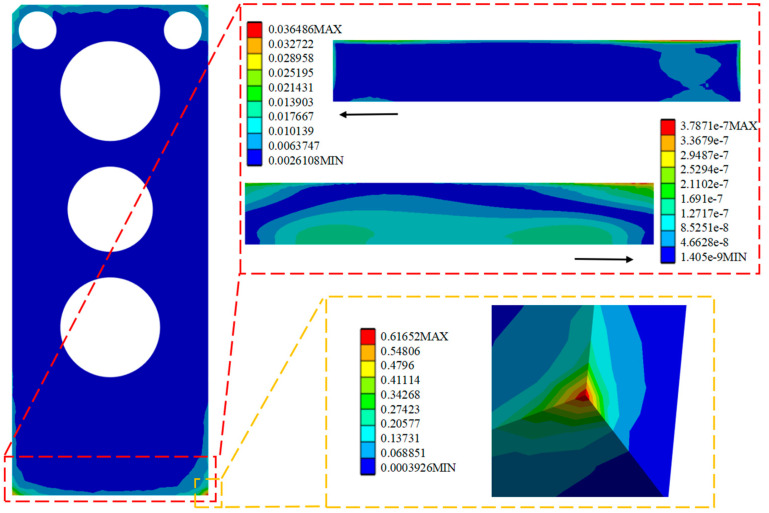
Apply acceleration excitation along the X-axis direction to the model before optimization.

**Figure 14:**
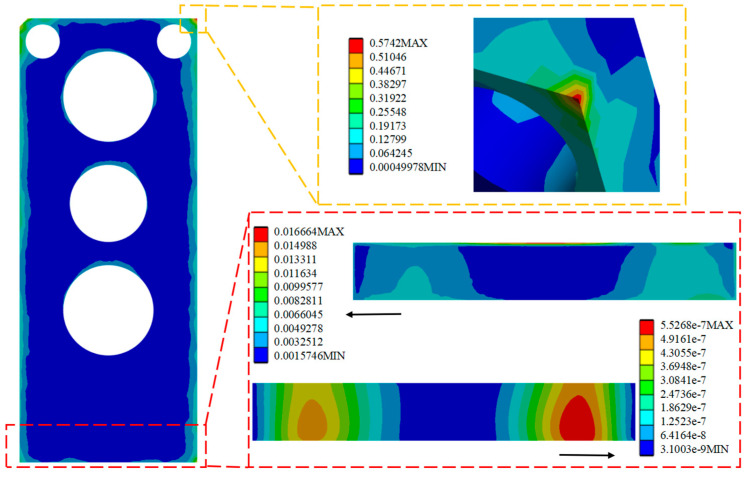
Apply acceleration excitation along the Y-axis direction to the model before optimization.

**Figure 15:**
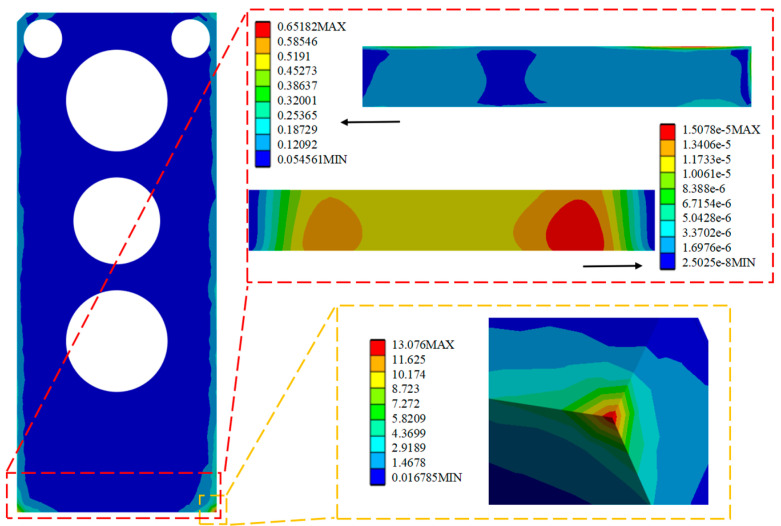
Apply acceleration excitation along the Z-axis direction to the model before optimization.

Through the analysis and solution of random vibration, the stress and strain cloud diagrams under the HPLD as a whole and the SL 3σ before optimization are shown in Figure 13, Figure 14, and Figure 15, respectively, in which the red box selects the enlargement for the stress–strain distribution cloud diagram of the SL, and the yellow box selects the enlargement for the HPLD at the maximum stress. As a result, the overall module equivalent force of the LD is prominent in the HPLD and module shell contact corners, easy to occur stress concentration; acceleration excitation is applied in the Z-axis direction, resulting in the equivalent force being much larger than the other two axes and its equivalent force value of 13.076 MPa. Analyzing the stress–strain distribution of the SL, the maximum stress of the SL is located in the SL at the edge of the SL, and its equivalent force under the 3σ are, respectively, 0.0365 MPa, 0.0167 MPa, and 0.652 MPa, and the acceleration excitation is applied in the direction of the Z-axis, which produces the equivalent stresses that are also much larger than the other two axes but much smaller than its yield limit. Only elastic strain occurs in the SL under this random vibration load, and its fatigue mode is high-cycle fatigue.

**Figure 16:**
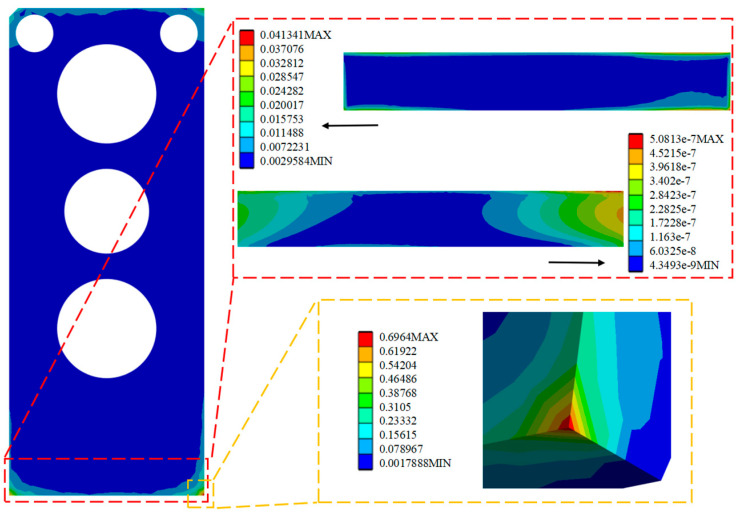
Apply acceleration excitation along the X-axis direction to the optimized model.

**Figure 17:**
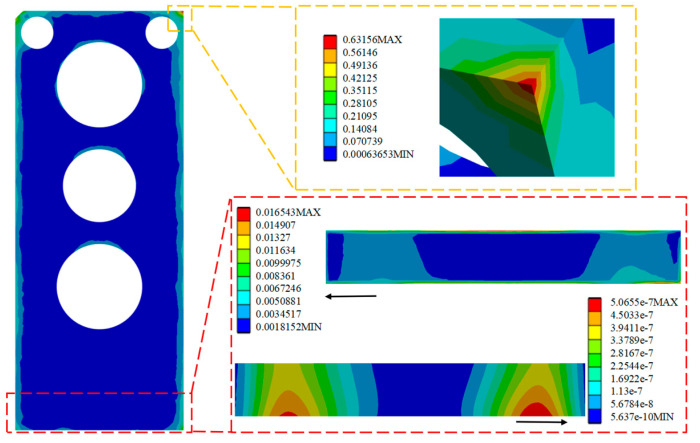
Apply acceleration excitation along the Y-axis direction to the optimized model.

**Figure 18:**
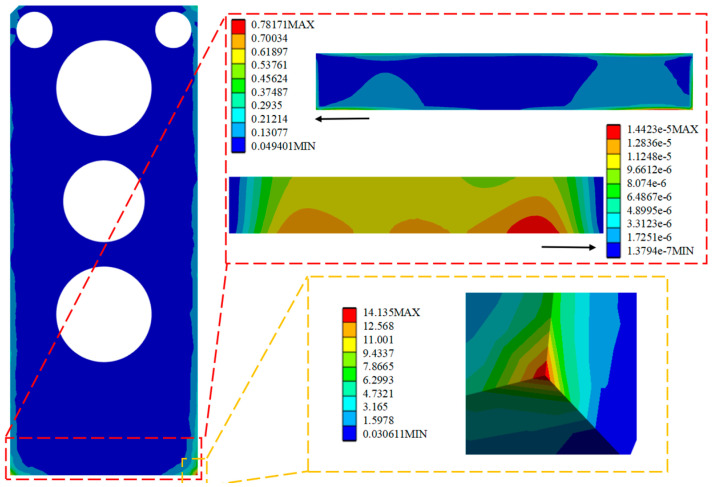
Apply acceleration excitation along the Z-axis direction to the optimized model.

By analyzing and solving the random vibration, the optimized stress and strain maps under the HPLD as a whole and the SL 3σ are shown in Figure 16, Figure 17, and Figure 18, respectively, in which the red box selection zooms in on the stress–strain distribution map of the SL, respectively, and the yellow box selection zooms in on the maximum stress of the HPLD. It is concluded that the overall module equivalent stress of the LD occurs prominently at the contact corner of the HPLD and the module housing. Acceleration excitation is applied to the Z-axis direction, the equivalent force is much larger than the other two axes, and its equivalent force value is 14.135 MPa. Analyzing the stress–strain distribution of the SL, the maximum stress of the SL is located at the edge of the SL, and the equivalent force under its 3 is 0.0413 MPa, 0.0165 MPa, and 0.782 MPa, respectively, and the equivalent force under acceleration excitation is applied to the Z-axis direction, and the equivalent force is the same as that in the Z-axis direction. The equivalent force generated is also much larger than the other two axes but much smaller than its yield limit. Only elastic strain occurs in the SL under this random vibration load, and its fatigue mode is high-cycle fatigue.

### 4.3. Fatigue Life Prediction Under Vibratory Loading of the SL

The strain values of the SL before and after optimization can be obtained from the finite element strain analysis results, respectively. Under the conditions of 1σ, 2σ, and 3σ strain levels, the corresponding number of fatigue cycles can be calculated as shown in Table 7 and Table 8, where X, Y, and Z indicate the acceleration excitation applied to the X, Y, and Z axes directions, respectively.

Calculate the cumulative number of vibration cycles at each strain level before and after optimization, respectively:

(1) Before optimization

$$n_{1}=2261.2\times 3600\times 0.6831\approx 5560652.59$$

$$n_{2}=2261.2\times 3600\times 0.2710\approx 2206026.72$$

$$n_{3}=2261.2\times 3600\times 0.0433\approx 352475.856$$

(2) After optimization

$$n_{1}=2265.3\times 3600\times 0.6831\approx 5570735.15$$

$$n_{2}=2265.3\times 3600\times 0.2710\approx 2210026.68$$

$$n_{3}=2265.3\times 3600\times 0.0433\approx 353114.964$$

The fatigue life of the SL under random vibration conditions is predicted as follows:

$${T_{v}=1/D}_{v}=1/\left(\frac{n_{1}}{N_{1}}+\frac{n_{2}}{N_{2}}+\frac{n_{3}}{N_{3}}\right)$$

The solution is obtained:${\;T}_{v}$ = 1.24 × 10^21^ h.

Similarly, the predicted values of fatigue life under other conditions $T_{v}$ is shown in Table 9.

As can be seen from Table 8, the predicted lifetime of the SL of the HPLD under random vibration load in the Z-axis direction is much smaller than that in the other two axes. The life prediction study of the HPLD under random vibration load should focus on the Z-axis direction, and the life of the sSL under random vibration load applied in the Z-axis direction should be taken as the life of the SL under random vibration load. As a result, the lifetimes of the SL under random vibration loads in the package form before and after the use of optimization are 5.75 × 10^7^ and 8.31 × 10^7^ h, respectively, which are in accordance with the specified requirements.

## 5. Simulation Evaluation of the SL Reliability Under Thermal Vibration Coupled Loading

From the above, it can be seen that the thermal fatigue life of the SL before the optimization is 2851 cycles, and the individual thermal cycle period is 18,720 s. Under the 1 h composite loading (thermal–vibration coupling) condition, the thermal cycle fatigue damage factor can be deduced as

$$D_{th}=\frac{1}{2851\times 18720/3600}=0.00006745$$

The total damage factor is calculated by considering both thermal cycling fatigue and random vibration fatigue:

$$D_{total}=D_{th}+D_{v}=0.00006745+0.0000000174=0.00006747$$

The fatigue life of the SL under thermal vibration load loading is

$$T_{total}=\frac{1}{D_{total}}=14821\;h$$

Similarly, the fatigue life of the optimized SL under thermal vibratory loading is 29,786 h.

In the study of the cumulative effect of damage, comparing the damage factor of the SL under thermal cyclic loading and under random vibration loading, the thermal fatigue damage factor is significantly higher than the vibration damage. It can be seen that the fatigue life of the HPLD’s SL under thermal vibration load is mainly affected by the thermal cyclic load, and the life decay mainly originates from the microcrack expansion mechanism dominated by thermal cycling.

## 6. Conclusions

In this paper, the reliability study of thermal cycling, random vibration, and thermal vibration coupling is carried out for the SL of the HPLD, and the finite element model of the HPLD is established and solved. Under the two loading conditions of thermal cycling and random vibration, the fatigue failure of the SL is studied, and the maximum equivalent stress and strain distribution at this position are analyzed, and the fatigue life of the SL is predicted when the thermal vibration coupling load is applied together, and the main conclusions are as follows:

(1) When the thermal cycle load is loaded, the Anand viscoplastic intrinsic model is used to describe the mechanical properties of the SL, and it is found by observing the graphs that the dangerous areas of stress and strain on the SL before and after the optimization are located in the edge area of the SL; the difference in stress–strain in the X-axis direction of the SL is larger than that in the direction of the other axes; and the failure of the SL will be prioritized from the edge of the SL along the direction of the length of the cavity.
(2) The first six orders of vibration patterns and the intrinsic frequency of the LD module are obtained through modal analysis, and then the acceleration PSD load is applied to the LD module for random vibration analysis. The acceleration excitation is applied in the Z-axis direction, and the equivalent stress produced by the SL before and after optimization is much larger than that applied to the other two axes, but numerically, it is much smaller than its yield strength, and the SL only undergoes elastic strain and high circumferential fatigue under this random vibration load.
(3) The fatigue life prediction model under thermal cycling obtains the fatigue life of the hazardous area of the SL before and after optimization to be 2851 cycles and 5730 cycles, respectively; the fatigue life prediction model under random vibration load obtains the fatigue life of the hazardous area of the SL to be 5.75 × 10^7^ h and 8.31 × 10^7^ h, respectively; the fatigue life of the superposition of the damage of thermal vibration load obtains the fatigue life of the SL to be 14,821 h and 29,786 h, respectively. 29,786 h, and the damage under thermal cyclic loading is dominant.

The lifetime of the SL in the optimized package structure is greater than the lifetime of the SL before optimization, which is mainly due to the increase in fatigue lifetime of the SL during thermal cyclic load loading. This result is inextricably linked to the pre-optimization of moving the bar back to improve the heat dissipation path and increase the heat dissipation efficiency.

Electronic products working in the space environment are subjected to more loads than just thermal cycling and random vibration, and although other loads have a lower chance of causing the failure of electronic products, they cannot be ignored. Radiation, impact, and other factors also affect the life of electronic products in the space environment, so the same needs to study and analyze these factors. Restricted by the experimental conditions and other restrictions, this paper is carried out through the finite element simulation software, and in order to reduce the calculation time, the LD model has been partially simplified, such as wanting to make the results closer to the actual situation, the need for more accurate and detailed modeling simulation and experimental verification. This paper focused solely on the analysis of conventional indium solder technology. Future research should prioritize investigating processing techniques for solder layers with varying thicknesses and different solder materials, such as hard solders (e.g., Au-Sn). The aim is to reduce stress while ensuring longevity, thereby providing additional options for the application of high-power diode lasers in extreme environments. In the actual thermal cycling and random vibration environment, thermal cycling and random vibration will interact with each other to accelerate the process of the SL failure, and the simple use of linear superposition is not a true and accurate prediction of the fatigue failure of the SL. How to establish a fatigue life model that can predict the fatigue life of the SL is the focus of subsequent research.

## Figures and Tables

**Table 1:** Material properties of each layer in the LD.

Materials	Density (Kg/m^3^)	Young’s Modulus (10^9^ Pa)	CTE (10^−6^ 1/K)	Poisson’s Ratio	Thermal Conductivity (W/(m·K))	Specific Heat Capacity (J/(kg·K))
Cu	8930	115	16.5	0.36	398	384.56
GaAs	5310	86	6.4	0.3	44	320
In	7290	11	31	0.45	82	233
Au	19,280	74	14.2	0.42	315	128.7
Polyimide	1300	3.1	25	0.34	0.15	1100

**Table 2:** Parameters of Anand model for indium.

Parameters	Numerical Value
$Q/J\cdot {mol}^{-1}$	78,124
ξ	49.97
m	0.300
$A/1\cdot s^{-1}$	2.33 × 10^8^
$h_{0}/MPa$	500
a	1
$S_{0}/MPa$	28.3
$\hat{S}/MPa$	28.3
n	0.005

**Table 3:** Grid sensitivity analysis.

Mesh Count	Maximum Stress (MPa)	Maximum Strain (mm)	Maximum Stress Error	Maximum Strain Error
5142	8.0845	0.00073495	9.52%	9.61%
10,689	8.7305	0.0007949	2.29%	3.12%
17,785	9.0162	0.00082052	0.91%	0.92%
26,385	8.935	0.00081309		

**Table 4:** Material parameters of the LD module housing.

Material	Density/kg·m^−3^	Young’s Modulus/GPa	Poisson’s Ratio	Shear Modulus/GPa	Bulk Modulus/GPa
6061-T6	2713	69.04	0.33	25.955	67.686

**Table 5:** First six natural frequency modes of the LD module.

Order/Hz	1st	2nd	3rd	4th	5th	6th
Frequency before optimization	2261.2	3271.4	3917.5	4984.7	5200.6	5587.1
Frequency after optimization	2265.3	3271.5	3916.1	4984.7	5199.7	5600.1

**Table 6:** Acceleration power spectral density.

Frequency/Hz	PSD
20	0.052 g^2^/Hz
20–50	+6 dB/octave
50–800	0.32 g^2^/Hz
800–2000	−6 dB/octave
2000	0.052 g^2^/Hz
Overall	20.0 grms

**Table 7:** Pre-optimization of the laser SL fatigue cycles.

	$N_{1}$	$N_{2}$	$N_{3}$
X	5.02 × 10^30^	1.56 × 10^28^	5.31 × 10^26^
Y	2.15 × 10^29^	6.68 × 10^26^	2.28 × 10^25^
Z	2.33 × 10^17^	7.23 × 10^14^	2.46 × 10^13^

**Table 8:** Post-optimization of the laser SL fatigue cycles.

	$N_{1}$	$N_{2}$	$N_{3}$
X	4.34 × 10^29^	1.35 × 10^27^	4.58 × 10^25^
Y	4.45 × 10^29^	1.38 × 10^27^	4.70 × 10^25^
Z	3.38 × 10^17^	1.05 × 10^15^	3.57 × 10^13^

**Table 9:** Pre- and post-optimization of the SL fatigue life.

	Pre-Optimization	Post-Optimization
X	1.24 × 10^21^	1.07 × 10^20^
Y	5.31 × 10^19^	1.10 × 10^20^
Z	5.75 × 10^7^	8.31 × 10^7^

## Data Availability

The original contributions presented in this study are included in the article; further inquiries can be directed to the corresponding authors.
